# Fetal Liver Volume Assessment Using Magnetic Resonance Imaging in Fetuses With Cytomegalovirus Infection^†^

**DOI:** 10.3389/fmed.2022.889976

**Published:** 2022-05-16

**Authors:** Ameth Hawkins-Villarreal, Ana L. Moreno-Espinosa, Raigam J. Martinez-Portilla, Karen Castillo, Nadine Hahner, Ayako Nakaki, Lucas Trigo, Olivier Picone, Nathalie Siauve, Francesc Figueras, Alfons Nadal, Elisenda Eixarch, Anna Goncé

**Affiliations:** ^1^BCNatal - Fetal Medicine Research Center (Hospital Clínic and Hospital Sant Joan de Déu), Universitat de Barcelona, Barcelona, Spain; ^2^Institut d’Investigacions Biomèdiques August Pi i Sunyer (IDIBAPS), Barcelona, Spain; ^3^Fetal Medicine Service, Department of Obstetrics, Hospital “Santo Tomás”, University of Panama, Panama City, Panama; ^4^Iberoamerican Research Network in Obstetrics, Gynecology and Translational Medicine, Mexico City, Mexico; ^5^Department of Gynaecology and Obstetrics, Hôpital Louis Mourier, Hôpitaux Universitaires Paris Nord, APHP, Université Paris Diderot, Paris, France; ^6^Department of Radiology, Hôpital Louis-Mourier, Hôpitaux Universitaires Paris Nord, APHP, Université Paris Diderot, Paris, France; ^7^Centre for Biomedical Research on Rare Diseases (CIBERER), Barcelona, Spain; ^8^Department of Clinical Pathology, Hospital Clinic, University of Barcelona, Barcelona, Spain

**Keywords:** magnetic resonance imaging, fetal liver, pregnancy, fetal cytomegalovirus infection, fetal brain abnormalities

## Abstract

**Objective:**

To assess fetal liver volume (FLV) by magnetic resonance imaging (MRI) in cytomegalovirus (CMV)-infected fetuses compared to a group of healthy fetuses.

**Method:**

Most infected cases were diagnosed by the evidence of ultrasound abnormalities during routine scans and in some after maternal CMV screening. CMV-infected fetuses were considered severely or mildly affected according to prenatal brain lesions identified by ultrasound (US)/MRI. We assessed FLV, the FLV to abdominal circumference (AC) ratio (FLV/AC-ratio), and the FLV to fetal body volume (FBV) ratio (FLV/FBV-ratio). As controls, we included 33 healthy fetuses. Hepatomegaly was evaluated post-mortem in 11 cases of congenital CMV infection. Parametric trend and intraclass correlation analyses were performed.

**Results:**

There were no significant differences in FLV between infected (*n* = 32) and healthy fetuses. On correcting the FLV for AC and FBV, we observed a significantly higher FLV in CMV-infected fetuses. There were no significant differences in the FLV, or the FLV/AC or FLV/FBV-ratios according to the severity of brain abnormalities. There was excellent concordance between the fetal liver weight estimated by MRI and liver weight obtained post-mortem. Hepatomegaly was not detected in any CMV-infected fetus.

**Conclusion:**

In CMV-infected fetuses, FLV corrected for AC and FBV was higher compared to healthy controls, indicating relative hepatomegaly. These parameters could potentially be used as surrogate markers of liver enlargement.

## Introduction

Cytomegalovirus (CMV) is the most common congenital infection and is a major cause of sensorineural hearing loss and neurodevelopmental abnormalities worldwide (1). As maternal screening of infection is not recommended, its detection during pregnancy is usually achieved when sonographic signs suggestive of infection are found during routine scans (2). After confirmation of fetal infection, the combination of serial targeted ultrasound (US) examinations and magnetic resonance imaging (MRI) as a complementary tool for brain assessment have shown good diagnostic performance for determining symptomatic status at birth (3–7). In infants with symptomatic disease, the manifestations can range from unspecific-mild to multi-systemic involvement, with a particular predilection toward the reticuloendothelial system, especially the liver (8, 9). Although the brain is a major target of congenital CMV-infection (10), there is a lack of information on hepatic involvement in these fetuses. Traditionally, estimation of fetal liver size has been based on liver length measurement performed by US (11–13), however, there is no standardized imaging methodology for the assessment of hepatomegaly.

The aim of this study was to compare fetal liver volume (FLV) in CMV-infected with that of healthy fetuses. In addition, we compared FLV according to the severity of fetal brain abnormalities in infected fetuses and correlated the liver weight estimated by MRI with that found in cases with termination of pregnancy (TOP).

## Materials and Methods

This was a retrospective case-control study including consecutive pregnancies with a CMV-infected fetus in which prenatal MRI was performed for prognostic assessment over a 13-year period (July 2006-December 2019) in BCNatal (Hospital Clínic and Hospital Sant Joan de Déu, Barcelona, Spain) and during a 4-year period (January 2015-September 2019) in Hôpital Louis-Mourier, Paris, France. The study was approved by the Institutional Review Board of the Hospital Clinic **(HCB/2017/0564)** and Hôpital Louis-Mourier **(CEERB-Paris Nord/2020-012)**.

Cytomegalovirus-infected fetuses were considered severely or mildly affected according to prenatal brain US/MRI findings observed in US/MRI, as described previously (14, 15) (Supplementary Table 1). Most cases were diagnosed with the presence of US abnormalities found during routine second or third trimester scans. The remaining cases were diagnosed after maternal CMV screening by patients’ physicians. Fetal CMV infection was confirmed with extraction of CMV DNA from amniotic fluid samples using the QIAsymphony system (Qiagen, Hilden, Germany). Chromosomal abnormalities and toxoplasmosis infection were ruled out at the time of the amniotic fluid study. The estimated fetal weight (EFW) at the time of the MRI was defined as that obtained by US no more than 2 weeks before or after the MRI. Small for gestational age (SGA) was defined as EFW by US below the 10th percentile. After fetal US/MRI, women were counseled about the prognosis of the newborn. TOP was discussed according to Spanish/French laws. In these case of TOP, routine post-mortem examination (macroscopic and microscopic) of the fetus and the placenta was performed after obtaining informed consent from the parents. The maceration status of the fetus was established using the Langley criteria (16). Organs, including the liver, were weighed on an electronic scale as part of the standard autopsy procedure. The time from delivery to autopsy was also recorded. In cases with a live newborn, congenital CMV was confirmed by a positive polymerase chain reaction (PCR) of a urine or saliva sample taken within the first 48 h of birth. The control group was made up of low-risk singleton pregnancies with fetuses of normal growth without structural abnormalities attended at BCNatal resulting in healthy newborns that were also a control group in a previous prospective cohort study on prenatal MRI (17, 18). All the pregnant women included agreed to participate and provided signed informed consent. Controls did not undergo any additional genetic or infection testing apart from routine blood exams during pregnancy.

### Magnetic Resonance Imaging Acquisition

**FIGURE 1:**
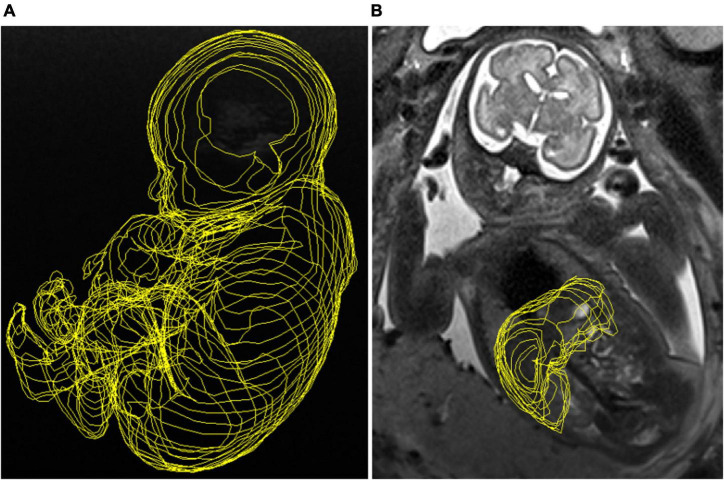
**(A)** Manual tracing of the total fetal body obtained from a T2-weighted imaging sequence in sagittal view. **(B)** Manual tracing image of the fetal liver volume obtained from a T2-weighted imaging sequence in coronal view.

Magnetic resonance imaging was performed in a clinical MRI system (1.5T Magnetom Aera syngo MR D13; Siemens, Erlangen, Germany in Hospital Clinic and 1.5T GE SIGNA Artist, Echo speed, LX MRI scanner, Milwaukee, WI, United States, in Hôpital Louis-Mourier) with a 5-channel cardiac coil in both centers. No maternal or fetal sedation was used. T2-weighted images were obtained using half Fourier single-shot turbo spin-echo (HASTE) sequences (Hospital Clinic: time of repetition [RT] = 1000 ms, echo time [ET] = 137 ms, slice thickness = 3.5 mm, gap = 3.5 mm, voxel size = 0.5859375 mm × 0.5859375 mm × 3.5 mm, field of view [FOV] = 225 mm × 300 mm, matrix = 256 mm × 192 mm, flip angle = 135°, acquisition time = 165 s. Hôpital Louis-Mourier: RT = 1120 ms, ET = 92 ms, slice thickness = 3.5 mm, gap = 4.0 mm, voxel size = 0.5469 mm × 0.5469 mm × 3.5 mm, FOV = 225 × 300, matrix = 256 mm × 320 mm, flip angle = 90°, acquisition time = 93 s) in axial, coronal, and sagittal planes according to fetal orientation. The total fetal body and liver area of the fetuses was assessed by manually tracing the region of interest on each image slice with tissue present using Fiji Image-J2 software. Figures 1A,B. Volumes were calculated using the sequence that allowed complete visualization of the fetus and the fetal liver without motion-induced artifacts. For the estimation of liver volume, the main portal vein, and gallbladder were excluded. The abdominal circumference (AC) was measured in the MRI using the same landmarks used for US measurement (19, 20). FLV was calculated in cubic centimeters (cm^3^) as follows: [(total liver area × pixel spacing^2^) × (slice thickness + GAP)]/1000. The total fetal body volume (FBV) in cubic centimeters (cm^3^) was calculated as: [(total body area × pixel spacing^2^) × (slice thickness + GAP)]/1000. We corrected the FLV for AC and for FBV (adapted from previously published methodology) (21, 22). To obtain the fetal liver weight estimated by MRI we used a literature-derived density of 1.05 g/cm^3^ from pediatric standards (23). We considered the GAP as a part of the formula to calculate the liver volume because this methodology was standard prior to 2020. The intensity of the liver signal in MRI analysis was not evaluated. MRI measurements were performed only at one center (BCNatal). Two of the physicians in charge of the MRI measurements were blinded to the diagnosis of fetal CMV-infection (KC, and LT) and the third knew the fetal CMV status (AH-V). The FLV was not assessed by US.

### Statistical Analysis

We compared the FLV, the FLV/FBV-ratio and the FLV/AC-ratio between CMV-infected and healthy fetuses and according to the severity of CMV infection. The variables were analyzed according to the severity of brain abnormalities in US/MRI imaging. Quantitative variables were assessed using the Shapiro–Wilk test for normality, and normally distributed variables were compared using the *t*-test and expressed as mean and standard deviation (SD). Non-normally distributed quantitative variables were compared using the Mann-Whitney *U*-test and expressed as median and interquartile range (IQR: p25–75). Qualitative variables were compared using the Chi squared (X^2^) and Fisher exact tests. A sub-analysis of variance and covariance (one-way ANOVA with Bonferroni *post-hoc* test) of the FLV, FLV/FBV-ratio and FLV/AC-ratio was performed according to a possible biological trend (healthy vs. mild vs. severe CMV-infected fetuses). Interobserver reliability analysis of FLV was performed comparing the measurements of AH-V and LT by a two-way random effect model to assess the intraclass correlation coefficient (ICC) estimates and their 95% confident intervals (95% CI) in 16 randomly chosen healthy fetuses. Concordance analysis between the fetal liver weight estimated by MRI and liver weight obtained post-mortem was made by a two-way random effect model to assess the ICC estimates and their 95% confidence intervals (CI) in those fetuses with anatomopathological examination.

Power estimation for an alpha error of 0.05 was performed using the two-sample means Satterthwaite’s *t*-test. A robust bias-corrected estimation was used to calculate the 95%CI and *p*-values. A *p*-value < 0.05 was considered significant. Data were analyzed using STATA, v.15.0 (College Station, TX, United States).

## Results

### Population Characteristics

A total of 31 pregnancies and 32 fetuses with CMV infection were included in the study (one monochorionic diamniotic pregnancy with both fetuses infected; one dichorionic diamniotic pregnancy with one fetus infected). The control group consisted of 33 singleton pregnancies. Twenty-two CMV-infected pregnancies were followed in the Fetal Infection Unit at the Hospital Clinic of Barcelona and 9 were followed in the Maternal-Fetal Medicine Unit at the Hôpital Louis-Mourier, Paris. Infection was confirmed by a positive PCR in amniotic fluid in 27 fetuses. Five patients at Hôpital Louis-Mourier declined amniocentesis, and infection was confirmed in urine of the newborns at birth. In 11 pregnancies fetal infection was suspected after maternal CMV screening in the first or second trimester. In 20 pregnancies, infection was suspected in the presence of fetal US anomalies: 13 during routine scans in the second trimester and 7 during the third trimester. Eighteen patients (58%) had a confirmed first trimester primary CMV infection, one had a second-trimester seroconversion, and in 12 (39%) the type of maternal infection was unknown. The mean gestational age (GA) at diagnosis was 26.4 (4.5) weeks. Of the total sample of CMV-infected fetuses, one showed no abnormal US/MRI findings, 16 fetuses had non-severe features of infection, and 15 had severe brain abnormalities. The most frequent US/MRI findings were periventricular hyperechogenicity (“halo sign”), ventriculomegaly, abnormal gyration and white matter hyperintensity in 71, 29, 43, and 67% of the fetuses, respectively. The most frequent extra-CNS findings were SGA and hyperechogenic bowel in 70 and 35% of the cases. The prenatal US/MRI findings are shown in Supplementary Table 3.

**FIGURE 2:**
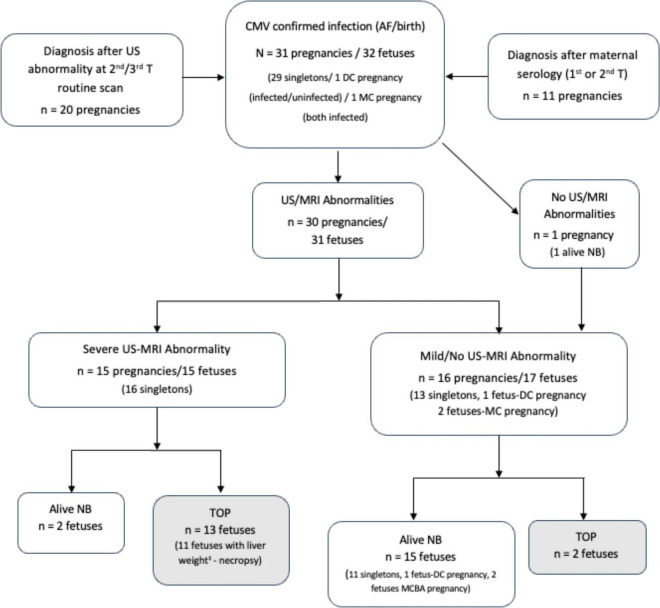
Flowchart: Follow-up of pregnancies with confirmed fetal CMV infection. Description of outcomes according to the severity of infection, anatomopathological examination performed and liver weight at necropsy^‡^. DC, dichorionic; MC, monochorionic; AF, amniotic fluid; US, ultrasound; MRI, magnetic resonance imaging; TOP, termination of pregnancy; NB, newborn.

Among the severely affected pregnancies, 13 women opted for TOP and post-mortem study was accepted by 11 (Figure 2: **Flowchart**). The median time from delivery to anatomopathological examination was 48 h. The median (IQR) of GA post-mortem was 30.3 (28.0–34.5) weeks. The median time (IQR) from MRI to post-mortem examination in cases with TOP was 1.0 (0.81–1.4) weeks. Eighty-two percent of the TOP-fetuses had none to mild maceration status. The median (IQR) of liver weight at post-mortem was 88.6 g (59.8–139).

The median GA (IQR) at MRI in the study population was 31.6 (28.6–33.4) weeks. The characteristics of pregnancies with and without CMV infection and according to the severity of brain abnormalities are summarized in Tables 1, 2. We observed a significantly higher proportion of pregnant women with a child less than 3 years old of age, SGA at US and at birth among CMV-infected fetuses Table 1. We also found a tendency to low educational level among mothers with pregnancies involving severe CMV-infected fetuses, *p* = 0.05, Table 2.

**TABLE 1:** Baseline characteristics in pregnancies with and without congenital CMV infection.

Characteristic	cCMV *n* = 31 preg/32 fetuses	Controls** *n* = 33 preg/fetuses	p* value
Maternal age, years, mean (SD)	32.4 (5.1)	32.8 (5.6)	0.86
Ethnicity (Caucasian), (%)	81	73	0.46
Multiparity, (%)	58	42	0.13
Low educational level, (%)	30	27	0.81
Child at nursery (<3 year child), (%)	**62**	**33**	0.01*
Fetal gender (female), (%)	62	51	0.37
Gestational age at MRI, mean (SD)	31.2 (2.7)	30.7 (3.0)	0.46
EFW (g) at the time of MRI, mean (SD)	1570 (649)	1845 (519)	0.11
Small for gestational age, (%)	**34**	**5**	0.012*
AC at the time of MRI (mm), mean (SD)	259.3 (38.1)	271.1 (42.2)	0.24
Fetal body volume (cm^3^), mean (SD)	2371.2 (889)	2588.2 (897)	0.34
Gestational age at birth, mean (SD)	**37.6 (3.16)**	**39.3 (1.25)**	0.025*
Birthweight (grams)^†^, mean (SD)	**2706 (822)**	**3326 (369)**	0.002*

Data are presented as mean and standard deviation (SD), standard error of the mean (SEM), frequencies or percentage (%). *p-value as determined with the t-test, Mann-Whitney U, X^2^ or Fisher’s exact test. cCMV, congenital cytomegalovirus; Preg, pregnancies; MRI, magnetic resonance imaging; EFW, estimated fetal weight. **Controls: healthy fetuses. AC, abdominal circumference. MCDA twin pregnancy in cCMV cases.

*^†^Seventeen cCMV cases with a live newborn included. The bold values indicate significant differences.*

**TABLE 2:** Baseline characteristics of pregnancies with congenital CMV infection according to the severity of brain abnormalities.

Characteristic	Mild cCMV *n* = 16 preg/17 fetuses	Severe cCMV *n* = 15 preg/fetuses	p* value
Maternal age, years, mean (SD)	32.8 (4.9)	32.4 (5.4)	0.83
Ethnicity (Caucasian) (%)	80	81	0.93
Multiparity, (%)	63	73	0.52
Low educational level, (%)	**13**	**47**	**0.05**
Child at nursery (<3 year child), (%)	56	73	0.32
Fetal gender (female), (%)	53	69	0.61
Gestational age at diagnosis in AF, mean (SD)	25.2 (4.9)	27.6 (3.8)	0.18
Gestational age at MRI, mean (SD)	31.9 (1.9)	30.4 (3.0)	0.13
EFW (g) at the time of MRI, mean (SD)	1758 (694)	1402 (539)	0.10
AC^à^ at the time of MRI (mm), mean (SD)	270 (36.0)	246 (37.1)	0.10
Fetal body volume (cm^3^), mean (SD)	2482 (900)	2260 (896)	0.50

Data are presented as mean and standard deviation (SD), frequencies or percentage (%). *p-value as determined with the t-test, Mann-Whitney U, X^2^ or Fisher’s exact test. cCMV, congenital cytomegalovirus; Preg, pregnancies; AF, amniotic fluid. ^à^AC, abdominal circumference. One MCDA twin pregnancy in mild cases. The bold values indicate significant differences.

### Results of Magnetic Resonance Imaging Analysis:

**FIGURE 3:**
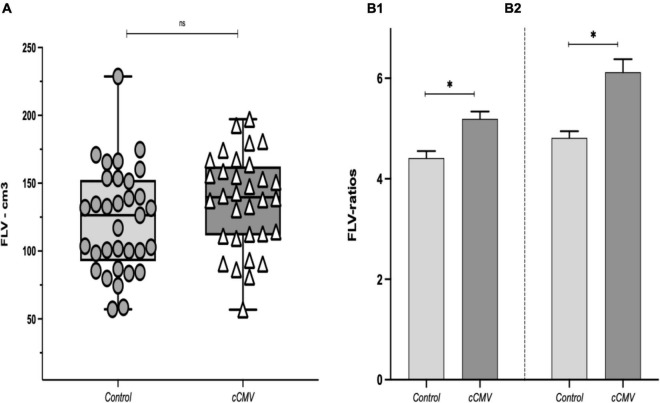
**(A)** Fetal liver volume (FLV) between study groups, data presented as median (IQR: interquartile range: p25–p75). [*p-value as determined with the Wilcoxon rank-sum test (Mann-Whitney U); ***p* = 0.22**]. **(B1)** FLV/AC-ratio (cm^3^/cm), and **(B2)** FLV/FBV-ratio between the study groups. Data presented as mean (SD). *p-value as determined with the *t*-test; ***p* < 0.001** in both. cCMV: congenital cytomegalovirus fetuses (*n* = 32). Controls: (*n* = 33). AC, abdominal circumference; FBV, fetal body volume.

We found no significant differences in FLV between CMV-infected fetuses and healthy-fetuses, (FLV [IQR]: 139.7 [112–161] vs. 126.4 [98–151], ***p* = 0.22**), Figure 3A. When the FLV was corrected for AC and FBV we observed significantly higher liver volumes in CMV-infected fetuses compared to the healthy controls [FLV/AC-ratio (SEM): 5.18 (0.14) vs. 4.41 (0.14), ***p* = < 0.001**; FLV/FBV-ratio (SEM): 6.11 (0.26) vs. 4.81 (0.13), ***p* = < 0.001**], Figure 3B1,B2.

**FIGURE 4:**
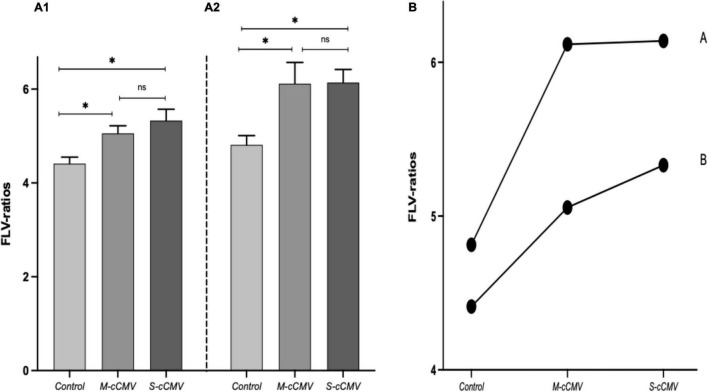
Comparison between the study sub-groups. Fetal liver volume (FLV) ratios. **(A1)** FLV/AC-ratio, **(A2)** FLV/FBV-ratio. Data presented as mean (SD). *p-value as determined with analysis of variance and covariance (one-way ANOVA); ***p* < 0.001**. **(B)** Linear trend analysis: FLV/FBV-ratio **(A)**, FLV/AC-ratio **(B)**. Data presented as mean (SD). *p-value as determined with parametric linear regression tendency test; ***p* < 0.001**. Control, healthy fetuses; M-cCMV, mild congenital cytomegalovirus fetuses; S-cCMV, severe congenital cytomegalovirus fetuses; ns, not statistically significant.

When the three study groups were compared, we also observed significantly higher ratios in the mild and in the severely infected cases compared to the healthy controls, Figure 4A. The FLV/AC-ratio and FLV/FBV-ratio remained higher when assuming a biological trend adjusted for GA at MRI, (***p* = < 0.001**) (parametric trend analysis), Figure 4B. However, we did not observe significant differences in the median liver volume or in the FLV/AC and FLV/FBV ratios according to the severity of CMV fetal brain abnormalities, Supplementary Figures 1A,B1,B2. The US/MRI findings, FLV, FLV/AC-ratio, FLV/FBV-ratio and liver weight at necropsy in severely affected fetuses with TOP and post-mortem examination are summarized in Supplementary Table 2. FLV showed a good correlation with the AC, GA at MRI, and FBV (*r* = 0.86, 0.82, 0.80, respectively; ***p* = < 0.001**). There was excellent agreement between examiners [ICC = 0.96 (95% CI: 0.90–0.98); ***p* = 0.01**; *n* = 16]. We also observed excellent concordance between fetal liver weight estimated by MRI and that obtained at anatomopathological examination [ICC = 0.95 (95% CI: 0.75–0.98): ***p* = < 0.001**]. There was no case of hepatomegaly in the post-mortem examination.

## Discussion

Our results show that hepatomegaly assessed by MRI is probably an uncommon finding in congenital CMV. Although there were no significant differences in FLV, when adjusted for fetal size (i.e., AC or FV-B) the FLV was increased in CMV-infected fetuses compared to healthy controls. We could hypothesize that this is due to liver enlargement in relation to body composition.

Although adjustment for AC and FBV is not a standard procedure and the diagnostic accuracy is not established, other authors have used ratios to better understand the pathophysiology of different fetal conditions. Cannie et al. demonstrated that the observed/expected fetal lung volume based on FBV could predict fetuses at high risk of pulmonary hypoplasia (21, 22). It can be argued that using the head circumference or cephalic diameter might have been a better normalization method in our study; however, microcephaly, a common feature of severe CMV infection, makes it less ideal.

As expected, one-third of the CMV-infected fetuses were SGA at the time of MRI and at birth (24, 25). The liver volume of these fetuses at MRI was similar to that of the control group. In this regard, Duncan et al. found that 63% of fetuses with an individualized birthweight <10th centile had a normal liver volume estimated by MRI (26). Interestingly, despite the larger hepatic ratios and greater number of SGA fetuses in the CMV-infected group in our sample, there were no statistically significant differences regarding AC measured at MRI. Despite the liver sizes, a lower AC would have been expected. There is controversy regarding the AC and liver size in intrauterine growth restriction (IUGR) fetuses. In this regard, Roberts et al. enrolled 98 fetuses with an AC below the 10th centile and found a liver length within normal limits in 82% (27). This may be because the measurement of AC may reflect not only liver size but also that of other intra-abdominal organs and the amount of fetal subcutaneous fat (28). Another possible explanation for the lack of significant differences in the AC is that compensatory arterial mechanisms may protect the liver from a reduction in blood flow in IUGR fetuses and thereby preserve the liver size in relation to the body composition (29, 30). In CMV infection the physiopathology of growth restriction and liver size could be related to mechanisms different from those in restricted fetuses due to placental dysfunction that could explain the finding of a relative liver enlargement in our cases.

Although we found a significantly higher FLV/AC-ratio and FLV/FBV-ratio in CMV cases compared to heathy controls, there were no significant differences in these ratios between mild and severely affected CMV-infected fetuses. We can speculate that perhaps mildly infected fetuses may already have hepatic involvement. Furthermore, hepatic involvement is not exclusively associated with severe fetal infection (31). Hepatic tropism and organ dysfunction in symptomatic CMV-infected newborns (9) is common. However, liver dysfunction as an isolated feature could be related to an acute phase of the infection in the second or third trimester and may not necessarily be related to adverse outcomes.

The mean liver weight in CMV-infected fetuses undergoing TOP at 30 weeks of gestation was 91.6 g. This was similar to the liver weights (53.4 to 98 g) of non-infected fetuses described by Maroun et al. at the same gestational age (32). This emphasizes our finding of no difference in the FLV estimated by MRI between CMV-infected and non-infected fetuses. As part of the internal validity of this study, we obtained excellent concordance between the FLV/weight estimated by MRI and the liver weight at post-mortem assessment. Liver weight is directly affected by the maceration status of the fetus (32, 33), being more affected in type-III maceration. However, 82% of the TOP-fetuses in our series had type-I maceration. Shelmerdine et al. found an excellent overall correlation between the liver volume estimated by MRI prior to autopsy and liver weight during autopsy (***r* = 0.98**) in 45 fetuses (60% with none to mild maceration) in a mean time of 10 days from delivery to autopsy (34). Other authors have also found an excellent correlation between the volume estimated by fetal MRI and post-mortem in different organs such as the brain (99% for cerebrum, 89% for cerebellum) supporting the reliability of MRI for weight assessment (35).

This study has some strengths and limitations that should be mentioned. One of the main strengths is the comparison of CMV-infected fetuses with healthy-controls, and between infected fetuses according to severity of infection. Moreover, the measurements were manually performed by the same single examiner in both centers, thereby reducing the variability. In addition, this is one of the first studies describing not only liver volume but also comparing the weight of the liver examined post-mortem with the liver weight estimated by MRI in CMV-infected fetuses undergoing TOP, providing information about the reliability of this measurement. Among the limitations, we first acknowledge the retrospective nature of the analysis. Second, the different MRI protocol between centers. Third, the small sample size in the infected group according to the severity of central nervous system abnormalities may preclude the finding of differences between these two groups and the overlapping of the results. Fourth, asymptomatic CMV infection was not discarded among healthy controls: however, this probability is very low since congenital infection occurs in 0.7% of all fetuses/newborns (1). Moreover, *in vivo* measurement of liver weight was obtained only in pregnancies undergoing TOP in the severely affected CMV group, limiting the interpretation of our results. Nevertheless, we considered these data as part of the internal validity of the study and despite only being available in a small proportion of fetuses, the data demonstrate the excellent correlation between the two measurements. Finally, although assessment of fetal volumes performed in this study provided additional data on the extent of the pathophysiology of congenital CMV-infection, its usefulness in clinical practice could be questioned. Moreover, measurement of FBV by MRI is time consuming (almost twice the time required for determining FLV) (23); nonetheless, as technology improves and semi-automatic delineation of fetal structures advances (36), the time to calculate volumes will likely be reduced.

## Conclusion

The liver volume obtained by MRI in CMV-infected fetuses was relatively greater than that of healthy-controls after adjustment for AC and FBV. Although increased FLV was not correlated with the severity of infection, these parameters could potentially be used as a surrogate marker of liver enlargement. Further studies are warranted to better understand the prognostic value of these findings.

## Data Availability Statement

The datasets generated and/or analyzed during the current study are not publicly available due to restrictions according to patient privacy regulations but are available from the corresponding author on reasonable request.

## Ethics Statement

The studies involving human participants were reviewed and approved by the Institutional Review Board of the Hospital Clinic (HCB/2017/0564) and Hôpital Louis-Mourier (CEERB-Paris Nord/2020-012). Written informed consent for participation was not required for this study in accordance with the national legislation and the institutional requirements. Written informed consent was obtained from the individual(s) for the publication of any potentially identifiable images or data included in this article.

## Author Contributions

EE, AH-V, and AG: conceptualization and design of the study. AH-V, ALM-E, EE, NH, ANak, OP, and NS: acquisition of data. ANad: anatomopathological examination. AH-V, RJM-P, AG, and EE: analysis and interpretation of data. AH-V, KC, and LT: MRI measurements. AG: supervision. AG, AH-V, and ALM-E: writing—original draft. AG, AH-V, EE, and FF: writing, revision, and editing of the submitted article.

## Conflict of Interest

The authors declare that the research was conducted in the absence of any commercial or financial relationships that could be construed as a potential conflict of interest.

## Publisher’s Note

All claims expressed in this article are solely those of the authors and do not necessarily represent those of their affiliated organizations, or those of the publisher, the editors and the reviewers. Any product that may be evaluated in this article, or claim that may be made by its manufacturer, is not guaranteed or endorsed by the publisher.

## Supplementary Material

Supplementary Figure 1 Fetal liver volume (FLV) and FLV-ratios according to the severity of brain abnormalities in CMV-infected fetuses (mild: *n* = 17, severe: *n* = 15). **(A)** FLV (cm^3^), data presented as median (IQR: interquartile range: p25–p75). **p*-value determined with the Wilcoxon rank-sum test (Mann-Whitney U); ***p* = 0.68**. **(B1)** FLV/AC-ratio. **p*-value as determined with the *t*-test; ***p* = 0.39**. **(B2)** FLV/FBV-ratio, data presented as mean (SEM, standard error of the mean).

## References

[1] Kenneson A Cannon MJ. Review and meta-analysis of the epidemiology of congenital cytomegalovirus (CMV) infection. *Rev Med Virol.* (2007) 17:253–76. 10.1002/rmv.535 17579921

[2] Guerra B Simonazzi G Puccetti C Lanari M Farina A Lazzarotto T Ultrasound prediction of symptomatic congenital cytomegalovirus infection. *Am J Obstet Gynecol.* (2008) 198:380.e1–7. 10.1016/j.ajog.2007.09.052 18191802

[3] Benoist G Salomon LJ Mohlo M Suarez B Jacquemard F Ville Y. Cytomegalovirus-related fetal brain lesions: comparison between targeted ultrasound examination and magnetic resonance imaging. *Ultrasound Obstet Gynecol.* (2008) 32:900–5. 10.1002/uog.6129 18991327

[4] Picone O Simon I Benachi A Brunelle F Sonigo P. Comparison between ultrasound and magnetic resonance imaging in assessment of fetal cytomegalovirus infection. *Prenat Diagn.* (2008) 28:753–8. 10.1002/pd.2037 18551722

[5] Birnbaum R Ben-Sira L Lerman-Sagie T Malinger G. The use of fetal neurosonography and brain MRI in cases of cytomegalovirus infection during pregnancy: a retrospective analysis with outcome correlation. *Prenat Diagn.* (2017) 37:1335–42. 10.1002/pd.5180 29119569

[6] Farkas N Hoffmann C Ben-sira L Lev D Schweiger A Kidron D Does normal fetal brain ultrasound predict normal neurodevelopmental outcome in congenital cytomegalovirus infection? *Prenat Diagn.* (2011) 31:360–6. 10.1002/pd.2694 21413035

[7] Lipitz S Hoffmann C Feldman B Tepperberg-Dikawa M Schiff E Weisz B. Value of prenatal ultrasound and magnetic resonance imaging in assessment of congenital primary cytomegalovirus infection. *Ultrasound Obstet Gynecol.* (2010) 36:709–17. 10.1002/uog.7657 20503234

[8] Boppana SB Ross SA Fowler KB. Congenital cytomegalovirus infection: clinical outcome. *Clin Infect Dis.* (2013) 57(Suppl. 4):178–81. 10.1093/cid/cit629 24257422 PMC4471438

[9] Bilavsky E Schwarz M Bar-Sever Z Pardo J Amir J. Hepatic involvement in congenital cytomegalovirus infection – infrequent yet significant. *J Viral Hepat.* (2015) 22:763–8. 10.1111/jvh.12374 25496231

[10] Cheeran MCJ Lokensgard JR Schleiss MR. Neuropathogenesis of congenital cytomegalovirus infection: disease mechanisms and prospects for intervention. *Clin Microbiol Rev.* (2009) 22:99–126. 10.1128/CMR.00023-08 19136436 PMC2620634

[11] Murao F Takamori H Hata K Hata T Kitao M. Fetal liver measurements by ultrasonography. *Int J Gynecol Obstet.* (1987) 25:381–5. 10.1016/0020-7292(87)90344-4 2889632

[12] Phatihattakorn C Ruangvutilert P Sansaneevithayakul P Boriboonhirunsarn D. Reference centile chart for fetal liver length of Thai fetuses. *J Med Assoc Thai.* (2004) 87:750–4. 15521228

[13] Tongprasert F Srisupundit K Luewan S Tongsong T. Normal length of the fetal liver from 14 to 40 weeks of gestational age. *J Clin Ultrasound.* (2011) 39:74–7. 10.1002/jcu.20756 21213331

[14] Hawkins-Villarreal A Moreno-Espinosa AL Eixarch E Marcos MA Martinez-Portilla RJ Salazar L Blood parameters in fetuses infected with cytomegalovirus according to the severity of brain damage and trimester of pregnancy at cordocentesis. *J Clin Virol.* (2019) 119:37–43. 10.1016/j.jcv.2019.08.008 31473566

[15] Leruez-Ville M Stirnemann J Sellier Y Guilleminot T Dejean A Magny JF Feasibility of predicting the outcome of fetal infection with cytomegalovirus at the time of prenatal diagnosis. *Am J Obstet Gynecol.* (2016) 215:342.e1–9. 10.1016/j.ajog.2016.03.052 27063062

[16] Langley FA. The perinatal postmortem examination. *J Clin Pathol.* (1971) 24:159–69. 10.1136/jcp.24.2.159 5551383 PMC476937

[17] Hahner N Benkarim OM Aertsen M Perez-Cruz M Piella G Sanroma G Global and regional changes in cortical development assessed by MRI in fetuses with isolated nonsevere ventriculomegaly correlate with neonatal neurobehavior. *Am J Neuroradiol.* (2019) 40:1567–74. 10.3174/ajnr.A6165 31467239 PMC7048445

[18] Hahner N Puerto B Perez-Cruz M Policiano C Monterde E Crispi F Altered cortical development in fetuses with isolated nonsevere ventriculomegaly assessed by neurosonography. *Prenat Diagn.* (2018) 38:365–75. 10.1002/pd.5240 29458235

[19] Salomon LJ Alfirevic Z Berghella V Bilardo C Hernandez-Andrade E Johnsen SL Practice guidelines for performance of the routine mid-trimester fetal ultrasound scan. *Ultrasound Obstet Gynecol.* (2011) 37:116–26. 10.1002/uog.8831 20842655

[20] Salomon LJ Alfirevic Z Da Silva Costa F Deter RL Figueras F Ghi T ISUOG practice guidelines: ultrasound assessment of fetal biometry and growth. *Ultrasound Obstet Gynecol.* (2019) 53:715–23. 10.1002/uog.20272 31169958

[21] Cannie MM Jani JC Van Kerkhove F Meerschaert J De Keyzer F Lewi L Fetal body volume at MR imaging to quantify total fetal lung volume: normal ranges. *Radiology.* (2008) 247:197–203. 10.1148/radiol.2471070682 18258812

[22] Cannie M Jani JC De Keyzer F Devlieger R Van Schoubroeck D Witters I Fetal body volume: use at MR imaging to quantify relative lung volume in fetuses suspected of having pulmonary hypoplasia. *Radiology.* (2006) 241:847–53. 10.1148/radiol.2413051228 17053198

[23] Breeze ACG Gallagher FA Lomas DJ Smith GCS Lees CC. Postmortem fetal organ volumetry using magnetic resonance imaging and comparison to organ weights at conventional autopsy. *Ultrasound Obstet Gynecol.* (2008) 31:187–93. 10.1002/uog.5199 18092338

[24] Mussi-Pinhata MM Yamamoto AY Brito RMM De Isaac ML De Carvalho E Oliveira PF Birth prevalence and natural history of congenital cytomegalovirus infection in a highly seroimmune population. *Clin Infect Dis.* (2009) 49:522–8. 10.1086/600882 19583520 PMC2778219

[25] Njue A Coyne C Margulis AV Wang D Marks MA Russell K The role of congenital cytomegalovirus infection in adverse birth outcomes: a review of the potential mechanisms. *Viruses.* (2021) 13:1–16. 10.3390/v13010020 33374185 PMC7823935

[26] Duncan KR Issa B Moore R Baker PN Johnson IR Gowland PA. A comparison of fetal organ measurements by echo-planar magnetic resonance imaging and ultrasound. *BJOG An Int J Obstet Gynaecol.* (2005) 112:43–9. 10.1111/j.1471-0528.2004.00318.x 15663396

[27] Roberts AB Mitchell JM McCowan LM Barker S. Ultrasonographic measurement of liver length in the small- forgestational-age fetus. *Am J Obstet Gynecol.* (1999) 180:634–8. 10.1016/S0002-9378(99)70266-8 10076140

[28] Bernstein IM Goran MI Amini SB Catalano PM. Differential growth of fetal tissues during the secong half of pregnancy. *Am J Obstet Gynecol.* (1997) 176:28–32. 10.1016/s0002-9378(97)80006-3 9024084

[29] Tchirikov M Schroder HJ Hecher K. Ductus venosus shunting in the fetal venous circulation: regulatory mechanisms, diagnostic methods and medical importance. *Ultrasound Obstet Gynecol.* (2006) 27:452–61. 10.1002/uog.2747 16565980

[30] Ebbing C Rasmussen S Godfrey KM Hanson MA Kiserud T. Redistribution pattern of fetal liver circulation in intrauterine growth restriction. *Acta Obstet Gynecol Scand.* (2009) 88:1118–23. 10.1080/00016340903214924 19707895

[31] Rawlinson WD Boppana SB Fowler KB Kimberlin DW Lazzarotto T Alain S Congenital cytomegalovirus infection in pregnancy and the neonate: consensus recommendations for prevention, diagnosis, and therapy. *Lancet Infect Dis.* (2017) 17:e177–88. 10.1016/S1473-3099(17)30143-3 28291720

[32] Maroun LL Graem N. Autopsy standards of body parameters and fresh organ weights in nonmacerated and macerated human fetuses. *Pediatr Dev Pathol.* (2005) 8:204–16. 10.1007/s10024-004-7084-0 15747100

[33] Shelmerdine SC Main C Hutchinson JC Langan D Sebire NJ Arthurs OJ. The use of whole body diffusion-weighted post-mortem magnetic resonance imaging in timing of perinatal deaths. *Int J Legal Med.* (2018) 132:1735–41. 10.1007/s00414-018-1906-5 30056622 PMC6208717

[34] Shelmerdine SC Chung KL Hutchinson JC Elliott C Sebire NJ Arthurs OJ. Feasibility of postmortem imaging assessment of brain: liver volume ratios with pathological validation. *Fetal Diagn Ther.* (2019) 46:360–7. 10.1159/000497158 30970374 PMC6979430

[35] Orasanu E Melbourne A Cardoso MJ Modat M Taylor AM Thayyil S Brain volume estimation from post-mortem newborn and fetal MRI. *NeuroImage Clin.* (2014) 6:438–44. 10.1016/j.nicl.2014.10.007 25379457 PMC4218943

[36] Kadji C De Groof M Camus MF De Angelis R Fellas S Klass M The use of a software-assisted method to estimate fetal weight at and near term using magnetic resonance imaging. *Fetal Diagn Ther.* (2017) 41:307–13. 10.1159/000448950 28355605

