# Supplementary material for: Fetal Liver Volume Assessment Using Magnetic Resonance Imaging in Fetuses With Cytomegalovirus Infection†
Source: Front Med (Lausanne). 2022 May 16;9:889976. doi: 10.3389/fmed.2022.889976 (PMC9150546; doi:10.3389/fmed.2022.889976)
Supplement: Supplementary file 1 [file Table_1.DOCX]

| **Supplementary table 1. Classification of prenatal US and MRI abnormalities in congenital CMV infection** | | |
| --- | --- | --- |
| Severe US/MRI brain abnormalities* | Mild US/MRI brain abnormalities** | Extra-cerebral US abnormalities** |
| Severe ventriculomegaly (≥ 15 mm) | Intraventricular adhesions | ^ Hyperechogenic bowel |
| Microcephaly (HC ≤ -3 SD) | Mild ventriculomegaly (10-14.9 mm) | ^^ Intrauterine growth restriction |
| Periventricular hyperechogenicity | Isolated Calcifications | † Hepatomegaly (right lobe ≥ p95) |
| Enlarged sub-arachnoid space (CCW>95^th^ centile) (Micrencephaly) | Calcifications of lenticulostriate vessels in basal ganglia | Intra-hepatic calcifications |
| Porencephaly (porencephalic cysts) | Sub-ependymal cysts | Pleural effusion |
|  | White matter hyperintensity (MRI) | Ascites |
| Agenesis/dysgenesis of corpus callosum |  | Pericardial effusion |
| Cerebellar/vermian hypoplasia (CTD <5^th^ centile) or cerebellar hemorrhage  Abnormal gyration / Cortical dysplasia (MRI) |  | Fetal hydrops  † Cardiomegaly (CTI ≥ 3.4 cm)  Oligohydramnios (DVP ≤ 2 cm)  Polyhydramnios (DVP ≥ 10 cm)  Placentomegaly ≥ 40 mm |

** Lesions of poor prognosis:* fetuses with at least one severe brain US or MRI abnormality ***Lesions of uncertain prognosis: fetuses with* mild brain US or MRI abnormalities or extra-cerebral US abnormalities exclusively

*US: Ultrasound. MRI: Magnetic Resonance Imaging. HC: head circumference; CCW: crania-cortical width; CTD: cerebellar transverse diameter;*

*DVP: deepest vertical pocket*

*^ Considered when the echogenicity of the bowel is equal or more intense than that of the fetal bones*

*^^ Considered when the estimated fetal weight is below the 10^th^ centile according to specific population tables with or without Doppler ultrasound anomaly.*

† *Hepatomegaly is considered when the right hepatic lobe length is ≥ p95 * Vol. 39, N0. 2, February 2011*

† *CTI:* *Cardiothoracic Index: considered abnormal if the heart is more than one-third of the thoracic diameter*

*Adapted from:* 1*- Leruez-Ville et al. Prognosis evaluation of fetal CMV infection. Am J Obstet Gynecol 2016;215:342.e1-9.*

2*- Goncé et al. TORCH and B19 Parvovirus infections during Pregnancy.* [*www.fetalmedbarcelona.org*](http://www.fetalmedbarcelona.org)
