# Supplementary material for: Fetal Liver Volume Assessment Using Magnetic Resonance Imaging in Fetuses With Cytomegalovirus Infection†
Source: Front Med (Lausanne). 2022 May 16;9:889976. doi: 10.3389/fmed.2022.889976 (PMC9150546; doi:10.3389/fmed.2022.889976)
Supplement: Supplementary file 2 [file Table_2.docx]

| Patient | GA  Diagnosis  US & AF | IgG/IgM^  IgG Avidity | US  CNS Findings | US  Extra-CNS  findings | MRI (GA)  CNS Findings | FLV (cm^3^) | FLV/AC  ratio | FLV/FBV  ratio | GA  TOP  w/d | Maceration  status ^ƒ^ | Liver weight (Necropsy) | Anatomopathological findings |
| --- | --- | --- | --- | --- | --- | --- | --- | --- | --- | --- | --- | --- |
| 1 | 22 w | Pos/Pos  Intermediate | PV hyperechogenicity  Anterior horns dilated  Enlarged sub-arachnoid  space | Hyperechogenic bowel  Hepatomegaly | **23.1:** Microcephaly  Mild delay in cortical development  Mild VMG | 49.7 | 3.14 | 7.46 | 24.0 | I | 44.0 g | Periventriculitis with sub-ependymal disruption and reactive gliosis. |
| 2 | 22 w | Pos/Pos  N/A | PV hyperechogenicity  PV calcifications  Thalami calcifications | Hyperechogenic bowel  IUGR  Cardiomegaly  Liver calcifications,  Oligohydramnios  Placentomegaly | **25.6:** Normal | 75.9 | 4.40 | 7.52 | 28.0 | I | 81.0 g | Cerebellum hypoplasia, CMV inclusions in brain, thyroid gland, lungs, liver, kidneys.  CMV chronic villitis. |
| 3 | 23 w | Pos/Neg  High | PV hyperechogenicity | IUGR | **24.2:** Hypoechogenic pinpoint images surrounding the lateral ventricles (posterior & temporal horn) | 56.7 | 3.00 | 6.17 | 25.1 | III | 20.1 g | IUGR, CMV inclusions in brain, liver, kidneys.  CMV chronic villitis. |

**Supplementary Table 2 (a)**. US/MRI findings, FLV, FLV/AC ratio, FLV/FBV ratio and liver weight in necropsy in severely affected fetuses with termination of pregnancy.

US: ultrasound. MRI: magnetic resonance Imaging. FLV: fetal liver volume. FLV/AC ratio: fetal liver volume to abdominal circumference ratio. FLV/FBV ratio: fetal liver volume to fetal body volume ratio. GA: gestational age in weeks (w). AF: amniotic fluid. CNS: central nervous system. N/A: not available. PV: periventricular. VMG: ventriculomegaly. IUGR: intra-uterine growth restriction. CC: corpus callosum. TCD: transverse cerebellar diameter. WM: White matter. IV: Intraventricular. IUGR: intrauterine growth restriction. ^ƒ^ Maceration: I: none/mild, II: moderate, III: marked.

| Patient | GA  Diagnosis  US & AF | IgG/IgM^  IgG Avidity | US  CNS Findings | US  Extra-CNS  findings | MRI (GA)  CNS Findings | FLV (cm^3^) | FLV/AC  ratio | FLV/FBV  ratio | GA  TOP  w/d | Maceration  status ^ƒ^ | Liver weight (Necropsy) | Anatomopathological findings |
| --- | --- | --- | --- | --- | --- | --- | --- | --- | --- | --- | --- | --- |
| 4 | 25 w | Pos/Neg  High | PV hyperechogenicity  CC & TDC <p5 | None | **28.0**:  Micrencephaly, Severe delay in cortical development. Right hemispheric atrophy | 109.4 | 4.97 | 5.97 | 29.0 | I | 88.6 | Polymicrogyria (occipital lobe, parietal lobe, frontal lobe). Delay in Silvio fissure development. Splenomegaly (spleen: 5.7 g). |
| 5 | 26 w | Pos/Pos  Intermediate | PV hyperechogenicity  PV cyst  Mild VMG | Hyperechogenic bowel | **30.4:**  Temporal lobe cyst**.**  Mild VMG | 137.3 | 5.86 | 5.96 | 34.6 | I | 140.3 g | Pachygyria - Polymicrogyria and cortical dysplasia (R-hemisphere). CMV inclusions: brain, kidneys & pancreas.  CMV placental villitis |
| 6 | 26.4 w | Pos/Neg  N/A | PV hyperechogenicity  PV calcifications  Delay in cortical maturation | IUGR | **27.5:**  Microcephaly. PV cysts,  WM hypersignal  Severe delay in cortical maturation (Lissencephaly) | 86.3 | 4.14 | 7.08 | 28.2 | I | 59.8 g | Micrencephaly  CMV inclusions: brain, lungs, liver, & kidneys.  CMV chronic villitis |

**Supplementary Table 2 (b)**. US/MRI findings, FLV, FLV/AC ratio, FLV/FBV ratio and liver weight in necropsy in severely affected fetuses with termination of pregnancy.

US: ultrasound. MRI: magnetic resonance Imaging. FLV: fetal liver volume. FLV/AC ratio: fetal liver volume to abdominal circumference ratio. FLV/FBV ratio: fetal liver volume to fetal body volume ratio. GA: gestational age in weeks (w). AF: amniotic fluid. CNS: central nervous system. N/A: not available. PV: periventricular. VMG: ventriculomegaly. IUGR: intra-uterine growth restriction. CC: corpus callosum. TCD: transverse cerebellar diameter. WM: White matter. IV: Intraventricular. IUGR: intrauterine growth restriction. ^ƒ^ Maceration: I: none/mild, II: moderate, III: marked.

**Supplementary Table 2 (c)**. US/MRI findings, FLV, FLV/AC ratio, FLV/FBV ratio and liver weight in necropsy in severely affected fetuses with termination of pregnancy.

| Patient | GA  Diagnosis  US & AF | IgG/IgM^  IgG Avidity | US  CNS Findings | US  Extra-CNS  findings | MRI (GA)  CNS Findings | FLV (cm^3^) | FLV/AC  ratio | FLV/FBV  ratio | GA  TOP  w/d | Maceration  status ^ƒ^ | Liver weight (Necropsy) | Anatomopathological findings |
| --- | --- | --- | --- | --- | --- | --- | --- | --- | --- | --- | --- | --- |
| 7 | 27 w | Pos/Neg  High | PV hyperechogenicity  Caudate nucleus cyst  Temporal lobe cyst  IV adhesion | Cardiomegaly | **29.2:** Mild delay in cortical maturation.  PV cyst, IV cysts (occipital horn)  Mild VMG  WM hypersignal | 130.5 | 5.39 | 5.58 | 30.3 | I | 103.6 g | CMV inclusions: lungs & liver.  CMV placentitis & villitis. |
| 8 | 29 w | Pos/Pos  High | Ventriculitis  Germinal matrix cyst  Mild unilateral VMG | None | **30.1:** Micrencephaly.  Polymicrogyria.  Encephalic asymmetry  Ventriculitis  Severe VMG | 111.1 | 4.28 | 4.65 | 31.1 | I | 76.5 g | Microcephaly. Encephalitis.  Polymicrogyria (frontal & temporal lobe – R hemisphere)  Extended ventriculitis with necrosis.  WM microglial nodes.  CMV inclusions in brain, lungs, liver, pancreas & kidneys. |
| 9 | 30 w | Pos/Neg  High | Cerebellar cysts | IUGR | **31.0:**  Cortical dysplasia, Polymicrogyria. Subdural hematoma. Cerebellum vermian cyst. Encephalomalacia | 158.0 | 6.38 | 7.85 | 32.0 | I | 111.0 g | Cerebellar hypoplasia & cortical hemorrhage.  Periventriculitis  Interstitial nephritis  CMV placentitis |

US: ultrasound. MRI: magnetic resonance Imaging. FLV: fetal liver volume. FLV/AC ratio: fetal liver volume to abdominal circumference ratio. FLV/FBV ratio: fetal liver volume to fetal body volume ratio. GA: gestational age in weeks (w). AF: amniotic fluid. CNS: central nervous system. VMG: ventriculomegaly. PV: periventricular. IUGR: intra-uterine growth restriction. CC: corpus callosum. TCD: transverse cerebellar diameter. WM: White matter. IV: Intraventricular. IUGR: intrauterine growth restriction. ^ƒ^ Maceration: I: none/mild, II: moderate, III: marked.

| Patient | GA  Diagnosis  US & AF | IgG/IgM^  IgG Avidity | US  CNS Findings | US  Extra-CNS  findings | MRI (GA)  CNS Findings | FLV (cm^3^) | FLV/AC  ratio | FLV/FBV  ratio | GA  TOP  w/d | Maceration  status ^ƒ^ | Liver weight (Necropsy) | Anatomopathological findings |
| --- | --- | --- | --- | --- | --- | --- | --- | --- | --- | --- | --- | --- |
| 10 | SeroConv  (8-14 w)  (AF: 33.1 w) | Pos/Pos  Low | 33 w:  Severe VMG  Temporal lobe cysts | None | **34.1:** Severe VMG  Subependymal cyst. TCD<p5  WM hypersignal | 168.0 | 6.20 | 4.85 | 34.5 | I | 139.0 g | Polymicrogyria. WM gliosis. Severe VMG. Occipital horn cysts, CMV inclusions in brain, lungs, liver, pancreas & kidneys.  CMV chronic villitis |
| 11 | 34 w | Pos/Neg  High | PV hyperechogenicity  Porencephalic cyst | None | **36.0**: WM hypersignal | 167.5 | 5.58 | 4.11 | 37.0 | II | 148.7 g | Focal encephalitis & gliosis, CMV inclusions in brain, lungs, liver, pancreas & kidneys  CMV chronic villitis |

**Supplementary Table 2 (d)**. US/MRI findings, fetal liver volume (FLV), FLV/AC ratio, FLV/FBV ratio and liver weight in necropsy in severely affected fetuses with termination of pregnancy.

US: ultrasound. MRI: magnetic resonance Imaging. FLV: fetal liver volume. FLV/AC ratio: fetal liver volume to abdominal circumference ratio. FLV/FBV ratio: fetal liver volume to fetal body volume ratio. GA: gestational age in weeks (w). AF: amniotic fluid. CNS: central nervous system. SeroConv: seroconversion. VMG: ventriculomegaly. PV: periventricular. IUGR: intra-uterine growth restriction. CC: corpus callosum. TCD: transverse cerebellar diameter. WM: White matter. IV: Intraventricular. IUGR: intrauterine growth restriction. ^ƒ^ Maceration: I: none/mild, II: moderate, III: marked.
