# Supplementary material for: Fetal Liver Volume Assessment Using Magnetic Resonance Imaging in Fetuses With Cytomegalovirus Infection†
Source: Front Med (Lausanne). 2022 May 16;9:889976. doi: 10.3389/fmed.2022.889976 (PMC9150546; doi:10.3389/fmed.2022.889976)
Supplement: Supplementary file 3 [file Table_3.DOCX]

| **Supplementary Table 3.** Prenatal US/MRI findings in CMV infected fetuses. | | | | | | |  |
| --- | --- | --- | --- | --- | --- | --- | --- |
| US findings |  | n (%) | MRI findings ^‡^ |  | n (%) |  |  |
| Severe cerebral US abnormalities (n=14) * |  |  | Severe cerebral MRI abnormalities (n=14) * |  |  |  |  |
|  | PV hyperechogenicity / "halo" | 10 (71) |  | Abnormal sulcation/gyration | 6 (43) |  |  |
|  | Severe VMG (≥ 15 mm) | 3 (21) |  | Microcephaly (HC ≤ -3 SD) | 5 (36) |  |  |
|  | CC hypoplasia / dysgenesis | 2 (14) |  | Severe VMG (≥ 15 mm) | 4 (29) |  |  |
|  | Abnormal sulcation/gyration | 2 (14) |  | Delayed cortical development | 4 (29) |  |  |
|  | Microcephaly (-3 SD)/ Micrencephaly | 1 (7) |  | CC Agenesis / dysgenesis | 2 (14) |  |  |
|  | Cerebellar hypoplasia | 1 (7) |  | Cerebellar hemorrhage/encephalomalacia | 1 (7) |  |  |
|  | Cerebellar hemorrhage/cysts | 1 (7) |  | Subdural hematoma | 1 (7) |  |  |
|  | Porencephaly | 1 (7) |  |  |  |  |  |
|  |  |  |  |  |  |  |  |
| Mild cerebral US abnormalities (n=7) ** |  |  | Mild cerebral MRI abnormalities (n=6) ^ƒ^ |  |  |  |  |
|  | Mild VMG (10-14.9 mm) | 3 (43) |  | White matter hyperintensity | 4 (67) |  |  |
|  | Periventricular calcifications | 2 (28) |  | Mild VMG | 3 (50) |  |  |
|  | Subependymal cysts | 1 (14) |  | Subependymal cysts | 1 (17) |  | |
|  | IV cysts | 1 (14) |  | IV cysts | 1 (17) |  |  |
|  |  |  |  | Periventricular calcifications | 1 (17) |  |  |
|  |  |  |  |  |  |  |  |
| Non-cerebral US abnormalities (n=20) *** |  |  |  |  |  |  |  |
|  | SGA | 14 (70) |  |  |  |  |  |
|  | Hyperechogenic bowel | 7 (35) |  |  |  |  |  |
|  | Cardiomegaly/ Pericardial effusion | 4 (20) |  |  |  |  |  |
|  | Placentomegaly | 3 (15) |  |  |  |  |  |
|  | Liver calcifications | 3 (15) |  |  |  |  |  |
|  | Ascites | 2 (10) |  |  |  |  |  |
|  | Hepatomegaly | 1 (10) |  |  |  |  |  |
|  | Splenomegaly | 1 (10) |  |  |  |  |  |
|  | Oligohydramnios (DVP< 2cm) | 1 (10) |  |  |  |  |  |
| Data are presented in frequencies or percentage (n, %). US: ultrasound. MRI: Magnetic Resonance Imaging. FBS: Fetal Blood Sampling. PV: Periventricular, CC: corpus callosum, VMG: ventriculomegaly,  IV: intraventricular, including temporal lobe. SGA: small for gestational age. DVP: deepest vertical pocket. HC: head circumference. WM: White Matter. Micrencephaly: (Enlarged subarachnoid space)  Fetuses could have more than one abnormality. Considering fetuses with severe brain abnormalities (n=14). * Considering fetuses with mild ultrasound abnormalities (n=8). ^ƒ^ Considering all fetuses with mild US/MRI findings (n=7). *** Considering all fetuses with non-cerebral US abnormalities (n=20). ^‡^ MRI was performed in 31 pregnant woman/32 fetuses, between 24 and 35 weeks of gestation: 21 with abnormal findings; 11 with normal MRI. | | | | | |  |  |
