# Supplementary figures and images for: Fetal Liver Volume Assessment Using Magnetic Resonance Imaging in Fetuses With Cytomegalovirus Infection†
Source: Front Med (Lausanne). 2022 May 16;9:889976. doi: 10.3389/fmed.2022.889976 (PMC9150546; doi:10.3389/fmed.2022.889976)

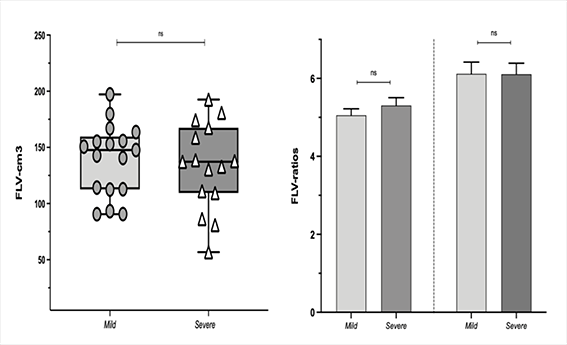

Supplement: Supplementary Figure 1 — Fetal liver volume (FLV) and FLV-ratios according to the severity of brain abnormalities in CMV-infected fetuses (mild: n = 17, severe: n = 15). (A) FLV (cm3), data presented as median (IQR: interquartile range: p25–p75). *p-value determined with the Wilcoxon rank-sum test (Mann-Whitney U); p = 0.68. (B1) FLV/AC-ratio. *p-value as determined with the t-test; p = 0.39. (B2) FLV/FBV-ratio, data presented as mean (SEM, standard error of the mean). [file Image_1.tiff]
